# Rethinking the Combination of Proton Exchanger Inhibitors in Cancer Therapy

**DOI:** 10.3390/metabo8010002

**Published:** 2017-12-23

**Authors:** Elisabetta Iessi, Mariantonia Logozzi, Davide Mizzoni, Rossella Di Raimo, Claudiu T. Supuran, Stefano Fais

**Affiliations:** 1 Department of Oncology and Molecular Medicine, National Institute of Health, Viale Regina Elena 299, 00161 Rome, Italy; elisabetta.iessi@iss.it (E.I.); mariantonia.logozzi@iss.it (M.L.); davide.mizzoni@iss.it (D.M.); rosella.diraimo@iss.it (R.D.R.); 2 Dipartimento Neurofarba, Sezione di Scienze Farmaceutiche, Laboratorio di Chimica Bioinorganica, Università degli Studi di Firenze, Polo Scientifico, Via U. Schiff 6, Sesto Fiorentino, 50019 Florence, Italy; claudiu.supuran@unifi.it

**Keywords:** acidity, hypoxia, pH, carbonic anhydrases, V-ATPases, proton pump inhibitors, carbonic anhydrase inhibitors

## Abstract

Microenvironmental acidity is becoming a key target for the new age of cancer treatment. In fact, while cancer is characterized by genetic heterogeneity, extracellular acidity is a common phenotype of almost all cancers. To survive and proliferate under acidic conditions, tumor cells up-regulate proton exchangers and transporters (mainly V-ATPase, Na^+^/H^+^ exchanger (NHE), monocarboxylate transporters (MCTs), and carbonic anhydrases (CAs)), that actively extrude excess protons, avoiding intracellular accumulation of toxic molecules, thus becoming a sort of survival option with many similarities compared with unicellular microorganisms. These systems are also involved in the unresponsiveness or resistance to chemotherapy, leading to the protection of cancer cells from the vast majority of drugs, that when protonated in the acidic tumor microenvironment, do not enter into cancer cells. Indeed, as usually occurs in the progression versus malignancy, resistant tumor clones emerge and proliferate, following a transient initial response to a therapy, thus giving rise to more malignant behavior and rapid tumor progression. Recent studies are supporting the use of a cocktail of proton exchanger inhibitors as a new strategy against cancer.

## 1. Introduction

**Figure 1:**
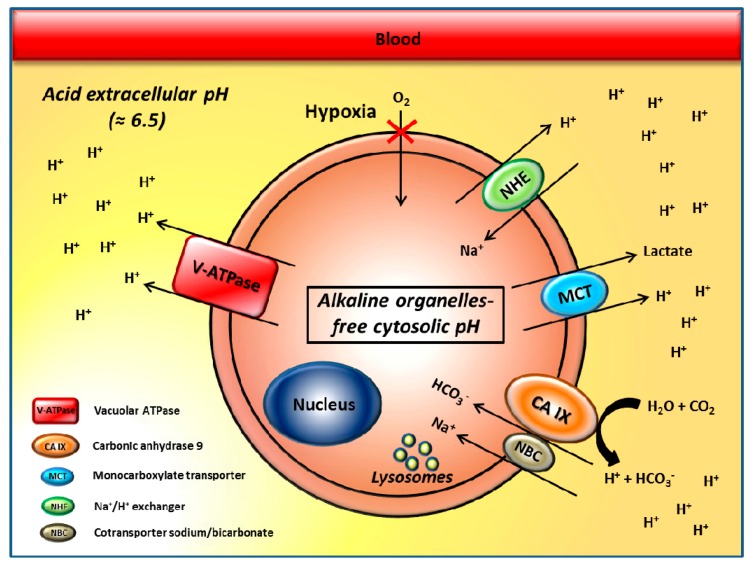
Proton flux regulators and their role in cancer. The aberrant expression and activity of proton exchangers leads to acidification of the tumor microenvironment and creates a reversed pH gradient across the plasma membrane leading to extracellular acidity and an alkaline, organelle-free cytosol.

Tumor cells often grow in a hypoxic microenvironment where there is low nutrient supply, and they upregulate glycolysis to sustain their high proliferation rate [1,2]. Growing evidence suggests that cancer cells take up much more glucose than normal cells, and mainly process it through aerobic glycolysis, the so-called “Warburg effect” [3,4]. This phenomenon leads to the conversion of one molecule of glucose into two molecules of lactic acid and 2 H^+^ to produce 2 ATP, compared to the 36 ATP produced by oxidative metabolism [1,2]. Thus, tumor cells implement glycolysis, promoting an abnormally high rate of glucose utilization, which in turn, leads to the accumulation of lactic acid and to the production of a large amount of H^+^ associated with proton efflux and extracellular pH reduction [5]. Associated with high glycolysis rate, high levels of carbonic dioxide produced during mitochondrial respiration of oxygenated cancer cells may also contribute to a substantial release of H^+^ into the tumor environment [6,7,8,9,10]. The complete oxidation of one glucose to carbonic dioxide yields 6 HCO_3_^−^ and 6 H^+^, leading to three times greater production of H^+^ than when glucose is converted to lactate, significantly accounting for tumor extracellular acidosis [6,7,8,9,10]. Uncontrolled growth, lactic and carbonic acid production, low blood and nutrient supply, contribute to the generation of a tumor microenvironment that is extremely toxic for either normal or more differentiated cells, and therefore, progressively selects cells able to survive in these adverse conditions. It is therefore conceivable that malignant cancer cells survive in this hostile microenvironment, thanks to the upregulation of the expression and activity of several proton extrusion mechanisms [11], which release protons and lactate into extracellular environment, avoiding the acidification of the cytosol. Among proton flux regulators are vacuolar H^+^-ATPases (V-ATPases), Na^+^/H^+^ exchanger (NHE), monocarboxylate transporters (MCTs), carbonic anhydrase IX (CA-IX) [11,12,13,14], and Na^+^/HCO_3_ co-transporters (NBC) [15] (Figure 1).

Two of the most studied proton flux regulators are vacuolar H^+^-ATPases (V-ATPase) [16,17,18] and carbonic anhydrase (CA) IX/XII [19,20,21,22]. The aberrant activity of these proton extruders creates a reversed pH gradient across the plasma membrane that is considered a hallmark of malignancy: extracellular acidity and alkaline conditions in the organelle-free cytosol, while normal cells show a neutral pH extracellularly, with a weakly acidic organelle-free cytosolic pH [23,24]. For this reason, the tumor pH gradient is called “reversed pH gradient” [5,16,25,26,27,28]. More precisely, the pH of tumor microenvironments have been shown to range between 6.0 and 6.8, with median values around 6.5, and the level of acidity was related with tumor malignancy [29,30,31,32]. Direct measurements of both intratumoral pO_2_ and pH revealed either a spatial heterogeneity between hypoxia and acidosis gradients [10,33,34], meaning that the areas of hypoxia and acidosis in tumors may not overlap in mouse tumor models, and a lack of correlation between CA-IX expression and various hypoxia markers [35,36,37]. In many tumors, chronic exposure to acidic pH has been reported to promote invasiveness, metastatic behavior, and resistance to cytotoxic agents [38,39,40,41,42]. All in all, tumor extracellular acidity is considered a crucial phenotype of malignant tumors, subjecting cancer cells to a sort of selective pressure, that independently from the tumor histotype, leads to the development of cells able to survive in such a hostile microenvironment. Notably, normal cells at pH ranging from very acidic to weakly acidic die or are entirely blocked in their functions [43,44]. For this reason, the acidic pH of solid tumors has been proposed as a therapeutic target and a drug delivery system for selective anticancer treatments [25,32,45]. Indeed, inhibition of these pH regulation systems has been reported to lead to potent antitumor effects [13]. Therefore, approaches aimed at inhibiting the proteins involved in pH regulation are now under exploitation for the design of novel promising alternative therapeutic anticancer strategies. NHE inhibitors were the first studied as interesting pharmacological agents for interfering with tumor hypoxia/acidosis [12]. Considering their significant toxicity and the lack of isoform-selectivity for other proteins involved in these processes (such as the MCTs) [46,47], the attention has been focused on the V-ATPases and CAs. Indeed, we have already reported that a specific inhibition of H^+^ release through proton pump inhibitors (PPIs) was able to induce acidification of the tumor cell cytosol [48], and acidic vesicle retention within tumor cells, with consequent increase in the antitumor activity of chemotherapeutic drugs [49], and significant antitumor effects [31,32,48,50]. Moreover, growing evidence in the literature is supporting the evidence that inhibition of CAs has potent antiproliferative and antimetastatic action [22,51]. A recent study has shown for the first time that the combination of proton pump and CA inhibitors (PPIs and CAIs) leads to a more efficient antitumor effect as compared to single treatments, representing the first attempt aimed at targeting, in a unique treatment, two important mechanisms involved in tumor acidification (i.e., tumor acidity and hypoxia) [52]. Herein, we will review the fields of the proton pump and CA inhibitors as promising agents in the management of solid tumors. This review will also attempt to emphasize the importance of using a cocktail of proton exchangers inhibitors as a new and innovative therapeutic strategy against cancer.

## 2. The pH Regulators Vacuolar H^+^-ATPases and Carbonic Anhydrase IX/XII

Tumor microenvironments are characterized by hypoxia, low blood supply, and acidity, which favor the generation of a microenvironment that is hostile and highly toxic for normal or more differentiated cells, inducing progressively, the selection against a more aggressive phenotype. Therefore, to avoid intracellular accumulation of toxic molecules, tumor cells upregulate the expression and activity of proton exchangers that maintain a relative neutral or even alkaline intracellular pH, through pumping protons into the extracellular environment or within the lumen of some membrane-bound organelles. Proton pump exchangers and CAs have been reported to be largely responsible for this hypoxic and acidic microenvironment [17,53,54].

Vacuolar-type ATPase (V-ATPase) is a ubiquitous proton pump shuttling protons from the cytoplasm towards intracellular organelles, and from inside to outside the cell plasma membrane [17,27,55,56]. It is a complex multi-subunit protein, composed of a transmembrane subunit, named V0 complex, devoted to proton transfer and a cytoplasmic portion, named V1 complex, that provides the necessary energy for proton translocation [55]. Its expression and activity are upregulated in many cancer cells. Augmented expression of V-ATPase is considered to be a well-designed compensatory mechanism that confers survival and growth advantages to cancer cells [57,58,59,60,61]. In tumor cells, the extrusion of protons by V-ATPases causes intracellular alkalinization and extracellular acidification, which are important mechanisms favoring the increased activation of extracellular metalloproteinases, thus contributing to tumor cell survival and growth, motility, invasion, metastasis, resistance to apoptosis, and multidrug resistance [62,63,64,65,66,67,68]. The V-ATPases are also expressed in vacuolar membranes (i.e., lysosomes), where they are involved in the transport of H^+^ into the lumen of intracellular organelles. Lysosomal acidification by V-ATPases has been reported to be under the control of lactate dehydrogenase B (LDHB), and to be facilitated by a physical interaction between LDHB and V-ATPase at the lysosomal surface [69]. Data obtained by Lu and collaborators [70] strongly demonstrated that inhibition of the V-ATPase has an antineoplastic activity [45]. In fact, the inhibition of V-ATPase function via knockdown of ATP6L expression using siRNA suppresses cancer metastasis by decreased proton extrusion, and downregulated protease activity [70]. Thus, this proton pump may be considered a suitable target for the development of novel anticancer strategy.

Carbonic anhydrases (CAs) are cellular pH regulators with a key role in the maintenance of pH homeostasis in cancer cells, thus representing suitable targets for anticancer therapies. CAs are a family of metalloenzymes that catalyze the reversible hydration of carbonic dioxide to bicarbonate and protons [22,71,72]. They are present in several tissues such us the gastrointestinal tract, the reproductive tract, the nervous system, kidney, lungs, skin, and eyes [20,22,73,74,75]. CAs are involved in respiration and acid–base equilibrium, electrolyte secretion, bone resorption, calcification, ureagenesis, gluconeogenesis, and lipogenesis [20,72]. Several isoforms are known, which are subdivided according to their location: membrane-bound/transmembrane, cytosolic, mitochondrial and secreted [21,22,72]. CA-IX and CA-XII are the two major tumor-related CA isoforms [76,77]. The two enzymes are transmembrane, multi-domain proteins formed of a short intra-cytosolic tail, a transmembrane short domain, an extracellular catalytic domain and a proteoglycan (PG)-like domain. CA-IX and CA-XII contribute significantly to the acidification of tumor microenvironment together with lactic acid production. Indeed, their inhibition has been reported to revert this phenomenon [19]. These two CA isoforms are predominantly found in hypoxic tumors with restricted expression in normal tissue, where they seem to be in their catalytically inactive state. Thus, their inhibition showed an anticancer effect with less side effects compared to other anticancer drugs [72,78]. For this reason, CA-IX and CA-XII have been considered attractive targets for cancer therapy, being validated recently as antitumor/antimetastatic targets [22,79].

Taking into account all these considerations, V-ATPases and tumor-associated CAs have been considered good candidates and ideal targets for the design of novel and innovative anticancer therapy, which interfere with tumor microenvironmental acidification, thus gaining a renewal interest in the last decade for oncologists.

## 3. V-ATPase Inhibitors

A bulk of evidence correlated hyperfunction of V-ATPases with tumor migration and invasion, multidrug resistance and metastatic processes. Consequently, inhibition of V-ATPase has become an attractive and promising strategy to counteract the tumor hypoxic and acidic microenvironment and to develop novel drugs for the benefit of cancer patients. Different anti-VATPases compounds have then been assessed as potential anticancer agents. Several studies are currently ongoing in order to better investigate in vitro and in vivo, in both preclinical and clinical settings, the binding properties and the mode of inhibition of V-ATPase inhibitors.

Among the V-ATPase inhibitors, the first identified and most frequently used are the natural compounds of microbial origin, bafilomycins, and concanamycins (belonging to plecomacrolide antibiotics) [80,81]. They are lipophilic compounds that have been reported to inhibit growth and to induce apoptosis in human cancer cells [59,82,83,84,85,86,87]. Other molecules capable of inhibiting V-ATPases via different mechanisms of action, such as benzolactone enamides, archazolid, and indolyls, were later discovered [88,89,90,91,92]. These compound, like bafilomycin A1 or concanamycin, have also investigated as anticancer agents [93,94]. Unfortunately, many reports evidenced high cytotoxicity of these ATPase inhibitors for normal cells, probably because V-ATPase is ubiquitously expressed and active in all types of cells, strongly hampering their potential clinical applications [16,45,55].

**Figure 2:**
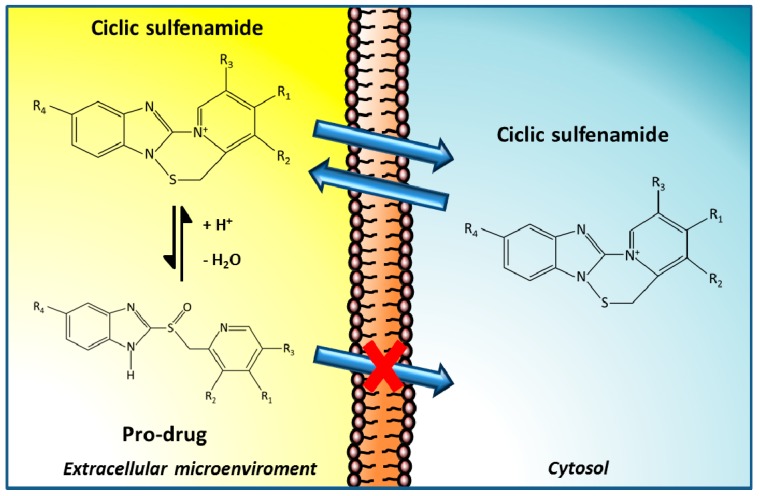
Proton pump inhibitors (PPIs) mechanism of action. PPIs are weak base pro-drugs, that once in the acidic extracellular environment surrounding tumors, can be protonated, thereby reducing their ability to cross the membrane of cells. PPIs then bind irreversibly to proton pumps, dramatically inhibiting their activity, leading to inhibition of proton translocation across the plasma membrane, which in turn, induces alkalization of the tumor microenvironment.

Our group focused the attention on another class of promising V-ATPase inhibitors, the family of proton pump inhibitors (PPIs), that include omeprazole, esomeprazole, lansoprazole, pantoprazole, and rabeprazole [95]. They are currently used as antiacid drugs against peptic diseases, including gastroesophageal reflux disease, peptic ulcers or functional dyspepsia [96]. These compounds are weak bases that need an acidic milieu in order to be transformed into the active molecule. Indeed, after protonation in the acidic spaces of the stomach, PPIs irreversibly bind to the cysteine residues of proton pump, dramatically inhibiting proton translocation and acidification of the extracellular environment [97] (Figure 2).

The specific targets of PPIs are H-ATPases contained within the lumen of gastric parietal cells, and through a lesser activity, they inhibit the activity of V-ATPases, thus blocking proton transport across membranes [12,13,45,95]. Some reports also refer to that PPIs could be useful in blocking ATPase activity in tumor cells. These agents did not show relevant systemic toxicity for normal cells, even in prolonged treatments and at very high dosages [98]. Therefore, PPIs have represented an attractive possibility as V-ATPases inhibitors, as they require an acidic environment to be activated, such as that found in the tumor microenvironment, which provides the possibility of tumor specific selectivity, and thus, of targeted anticancer strategy [56]. Therefore, several in vitro and in vivo studies have been carried out on PPIs, in both preclinical and clinical settings, for the design of novel anticancer therapy which target specifically tumor acidity.

### 3.1. PPIs as Therapeutic Agents

The first evidence supporting the use of PPIs in cancer was provided in 2004 when our group demonstrated that pre-treatment with PPIs (such as omeprazole, esomeprazole, or pantoprazole) in vitro and in vivo (i) reverted chemoresistance of different human tumor cell lines to cisplatin, 5-fluorouracile and doxorubicin; and (ii) increased the sensitivity of drug-sensitive cells to anticancer agents [49]. These effects were mediated by the intracellular retention of chemotherapeutic agents into the lysosomal organelles, associated with a “normalization” of the pH gradients of the tumor cells [49]. Additional studies investigated the response of B-cell lymphoma cell lines and acute lymphoblastic leukemia (ALL) bone marrow blasts, to omeprazole and esomeprazole. PPIs were able to induce a cytotoxic effect against both B-cell tumors and pre-B acute lymphoblastic leukemia cells obtained from patients with acute lymphoblastic leukemia (ALL). The cytotoxic effect was exerted through (i) activation of reactive oxygen species (ROS); (ii) perturbation of lysosomal membranes; (iii) alkalinization of acidic vesicles; (iv) acidification of the cytosol; (v) caspase-independent cell death [48]. PPIs were also able to significantly delay human tumor growth in SCID mice engrafted with B cell lymphomas [48]. Similarly, PPIs exerted a strong tumor inhibitory action against different human melanoma cell lines as well as in tumor-bearing mice [31]. Treatments with PPIs in different pH conditions, induced a marked cytotoxicity that was exerted through a caspase-dependent mechanism strongly influenced by low pH. In vivo studies in SCID mice engrafted with human melanoma pointed out that the tumor growth delay induced by PPIs was consistent with a clear reduction of pH gradients in tumor tissue [31]. We further demonstrated either in vitro and in vivo that pre-treatment with PPIs were able to improve the cytotoxic activity of suboptimal doses of the chemotherapeutic agent paclitaxel against human melanoma cells [32]. Then, we focused on identifying the most effective and promising agent within the PPI family, and discovered that lansoprazole was the most effective single agent against tumor cells [50]. Based on this finding, we investigated the efficacy of lansoprazole against multiple myeloma cell lines, showing that this drug was capable of inducing a strong tumor inhibitory action against this troublesome neoplasm, leading to a caspase-independent cell death [99]. Lastly, we showed that acidity represents a potent mechanism of tumor immune escape, and that PPIs increase the immune reaction against tumors [25,44,100]. Beside the direct toxic effects, PPIs were also able to inhibit mTOR signaling and other metabolic pathways in gastric carcinoma cell line, and to potentiate the antitumor effectiveness of Adriamycin in mice harboring gastric carcinoma xenografts [101,102,103,104,105,106]. In gastric cancer models, PPIs induced cancer stem cell depletion which passed through inhibition of proliferation, sphere formation, and 5-fluorouracil chemoresistance [107]. PPIs were also tested on breast cancers. Different studies demonstrated that pre-treatments with lansoprazole were able to potentiate the cytotoxic effect of doxorubicin, with no significant effect on non-neoplastic breast epithelial cells [108,109,110,111,112,113,114]. In esophageal cancer cell lines, esomeprazole significantly reduced cell viability, adhesion, and migration, as well as enhanced the cytotoxic effects of cisplatin and 5-fluorouracil [115]. Moreover, in ovarian carcinomas, omeprazole pre-treatments have been reported to induce a synergistic effect with paclitaxel on tumor growth in orthotopic and patient-derived xenograft mouse models [116]. Comparable results have been obtained by other groups that have been focused their investigations on hepatocellular, pancreatic, prostatic, and brain tumors [85,117,118,119,120,121,122].

Therefore, these results provided the proof of concept that PPIs may be considered, not only chemosensitizer agents, but also a new class of antineoplastic drugs, and described the background for a series of clinical studies aimed at supporting the use of PPIs as chemosensitizers.

### 3.2. PPIs in Clinical Trials

Up to now, two main clinical trials in humans investigated the effect of combined application of PPIs and chemotherapy treatment in cancer. The studies were performed in either osteosarcomas or metastatic breast cancer patient (MBC) [123,124]. The results showed that pre-treatment with PPIs increased the effectiveness of neoadjuvant chemotherapy in osteosarcomas patients, increasing the overall response rate [123]. Confirmation came with the second trial that enrolled women with metastatic breast cancer [124]. This phase II clinical trial aimed at evaluating whether esomeprazole improves efficacy of docetaxel and cisplatin treatment in metastatic breast cancer. The results showed that high dose PPIs proved beneficial and improved chemotherapy efficacy [124]. Indeed, patients that underwent esomeprazole treatment experienced the highest response rates and the longest survivals.

Another phase I clinical trial assessed the effectiveness and safety of the combined use of pantoprazole and doxorubicin in patients with advanced solid tumors, identifying the dose of 240 mg as the baseline for future phase II studies in patients with castration-resistant prostate cancer [125]. Moreover, a study on head and neck tumor patients confirmed that treatments with PPIs increase the overall survival of patients [126].

A more recent study on three patients affected with gastrointestinal cancer, refractory to standard chemotherapy, reported that high-dose of the PPI rabeprazole were able to sensitize human metastatic colorectal cancer to metronomic capecitabine, leading to a good quality of life with acceptable side effects [127]. This combined approach (rabeprazole with capecitabine) is currently under investigation in an approved clinical II trial, proving to be beneficial for the patients [128].

Lastly, two studies in companion animals with spontaneous tumors have been performed in order to explore the effect of PPIs treatment in combination with chemotherapy. Interestingly, PPIs were able to reverse chemoresistance in refractory tumors, both hematopoietic (lymphoma) or solid (melanoma and squamous cell carcinoma) [129]. Moreover, PPIs increased the efficacy of metronomic chemotherapy, independently from the tumor histotype or the animal species affected by cancer [130].

All these studies provided the first clinical evidence that PPIs pretreatment could be easily included into the standard protocols in clinical oncology with a clear benefit for patients having the less favorable prognostic factors. Indeed, pretreatment with PPIs, by inhibiting proton pumps, induced a decrease of the protonation of extracellular tumor environment, in turn allowing the chemotherapeutics to be fully effective, improving the effectiveness of either chemical and biological drugs against cancer. Thus, tumor alkalinization could improve the outcome of patients by counteracting tumor chemoresistance.

## 4. Inhibitors of Carbonic Anhydrase IX/XII

In recent years, the involvement of CA-IX/XII in generating the peculiar pH gradients of tumor cells is becoming more and more evident [12]. In fact, inhibition of the enzyme catalytic activity with fluorescent sulfonamides has been reported to be able to revert this phenomenon [19]. Moreover, accumulating experimental evidence recognizes that disruption of CA-IX by gene knockdown or inhibition of its catalytic activity with small molecules and/or antibodies strongly correlates with both extracellular and intracellular pH, tumor growth [78,131,132,133], tumor cell migration/invasion [134,135,136], chemo- or radiotherapy resistance [137,138].

As a consequence, different pharmacological inhibitors that specifically target the tumor-associated isoforms CA-IX and -XII were developed and tested for their antitumor activity during the last years [20].

### 4.1. CA-IX/XII Inhibitors as Therapeutic Agents

Many sulfonamide, sulfamate, sulfamide, and coumarine CA inhibitors were reported to efficiently target CA-IX [139,140,141]. The compounds specifically designed for targeting CA-IX were: (i) fluorescent sulfonamides, used for imaging purposes [19,142]; (ii) positively or negatively-charged compounds that inhibit selectively extracellular CAs [74]; (iii) nanoparticles coated with CAs [143]; (iv) monoclonal antibodies, among which M75 is a highly specific anti-CAIX mAb targeting the PG domain of CA-IX, discovered by Pastorekova’s group [144,145,146].

The most studied and important CA-IX/XII inhibitors are the sulfonamides, that exert their potent CA inhibitory properties by binding to the catalytic site of Cas, blocking then, its function [147,148]. Several inhibitors of the sulfonamide type have been identified for both CA-IX [76] and CA-XII [77]. Unfortunately, most of these compounds did not show specificity for the inhibition of the tumor-associated isoforms IX and XII versus the remaining CAs. Therefore, different sulfonamide derivates, more selective inhibitors of the tumor-associated CAs (CA-IX and XII) were designed and developed for targeting these agents, mainly through structure-based drug design approaches [149]. With the discovery of the X-ray crystal structure of CA-IX by the De Simone’s group in 2009 [150], the drug-design studies of CA inhibitors targeting isoform IX/XII were highly favored, and highly isoform-selective inhibitors were identified [139,140,141]. These compounds have been shown, over the past years, to be very promising in anticancer therapies. For instance, a fluorescent sulfonamide with high affinity for CA-IX was generated. This compound was used to determine the role of CA-IX in tumor acidification and for imaging purposes [19]. Indeed, Dubois et al. showed that fluorescent sulfonamides accumulate selectively in the hypoxic regions of xenograft animals with transplanted hypoxic colorectal cancers [142,151]. The in vivo proof of concept that sulfonamide CA-IX inhibitors may show antitumor effects has been first published by Neri’s group [152]. Similar studies from different laboratories on diverse models and cancer types demonstrated that sulfonamide CA-IX/XII inhibitors have a profound effect in inhibiting the growth of primary tumors, and great in vivo anti-metastatic effects [79,152,153,154,155,156]. These inhibitors were also able to induce depletion of cancer stem cells in models of breast cancer metastasis, leading, in turn, to diminished tumor growth and metastasis, and showing a promising efficacy for the recurrence of some cancers [157]. Supuran and coworkers developed a sulfonamide-derived compound, SLC-0111 (also known as WBI-5111), that strongly and specifically inhibits CA-IX, in vitro and in vivo [79,154]. This compound inhibits tumor growth and metastasis formation alone or in combination with antineoplastic drug, and decreased the cancer stem cell population in breast cancer models [79,154,158]. It has now completed the phase I clinical trials and is scheduled for phase II trials later this year [22,156].

Coumarins are another class of CA-IX and XII inhibitors. These natural compounds, together with the highly isoforms-selective CA-Is, are selective against the tumor-associated isoforms IX and XII at nanomolar concentrations, being ineffective against the broadly expressed isoforms CA-I and CA-II [153,159]. One of these derivatives, a glycosyl coumarin, was recently shown to inhibit the growth of primary tumors and the formation of metastasis in the highly aggressive 4T1 syngeneic mouse metastatic breast cancer [153]. These inhibitors are still under investigation in pre-clinical studies [160].

Monoclonal antibodies may represent another venue for the selective CA-IX and XII inhibition [144,161]. M75 is a highly specific anti-CAIX monoclonal antibody targeting the proteoglycan domain of CA-IX, discovered by Pastorekova’s group [78] and widely used in immunohistochemical and Western blot studies. WX-G250 (known also as girentuximab), the first CA-IX inhibitor to enter clinical trials [162], is another chimeric monoclonal antibody that is actually in phase III clinical trials, as an adjuvant therapy for the treatment of non-metastasized renal cell carcinoma. CA-XII specific monoclonal antibodies have also been generated and characterized. The first and most significant anti-CAXII monoclonal antibody (6A10) was created by Battke and coworkers [144]. The antibody binds to the catalytic domain of CA-XII, inhibiting its activity at nanomolar concentrations. 6A10 was shown to successfully inhibiting the growth of tumor spheroids in vitro and efficiently decreased tumor growth in vivo [144,163].

### 4.2. CA-IX Inhibitors in Clinical Trials

In the past few years, the tumor-associated cell surface CA isoform, CA-IX, has been validated as new anticancer drug target for the treatment and imaging of cancers expressing this enzyme, and some therapeutic strategies against CA-IX have already entered in clinical studies.

Serum levels of CA-IX have been explored as a potential biomarker for the treatment response in patients with metastatic renal cell cancer (mRCC) in a pilot human trial [164]. The study collected blood samples from 91 patients with mRCC and 32 healthy individuals, and associated serum CA-IX levels with the occurrence of tumor progression (stage, tumor grade, tumor size, recurrence, and metastasis), suggesting that CA-IX may be a valuable diagnostic and prognostic tool in RCC [164]. Serum CA-IX levels have also been suggested as a potential biomarker to predict the outcome for patients with mRCC [165]. Correlation between CA-IX level of expression and recurrence, survival, and clinical response to therapy was also observed in patients with high-risk, nonmetastatic renal cell carcinoma [166] and laryngeal cancer [167].

A selective small molecule, an ureido-substituted benzenesulfonamide derivative, SLC-0111, entered, in 2014, a phase I clinical trial for the treatment of advanced, metastatic solid tumors to evaluate the safety, tolerability, and pharmacokinetics of this compound (NCT02215850). Although the study was completed in March 2016, the results have not yet been published. 

A novel small molecule radiotracer, 18F-VM4-037, that binds to CA-IX in clinically localized kidney tumors, has been submitted to a phase II clinical trial sponsored by National Cancer Institute (NCI) (NCT01712685). The main objective of the study was to test the safety and effectiveness of 18F-VM4-037 during imaging studies of kidney cancer. The study enrolled 12 patients with renal cell carcinoma, and demonstrated that it is a well-tolerated agent which allows imaging of CA-IX expression both in primary tumors and metastases, highlighting 18F-VM4-037 as a useful drug in the evaluation of metastatic ccRCC lesions [168].

Regarding antibodies, the monoclonal antibody G250 was the first monoclonal antibody anti-CAIX introduced in a phase I/II clinical trials in combination with interferon-alpha-2a in metastatic renal cell carcinoma patients (mRCC), who are at a high risk of recurrence after resection of the primary tumor [162]. The study on 31 patients with mRCC treated with both drugs demonstrated that the treatment was safe, well tolerated, and led to clinical disease stabilization [162]. This antibody also progressed to a phase III clinical trial as an adjuvant therapy for the treatment of patients with metastatic renal cell carcinoma (NCT00087022). This study had proposed to investigate the efficacy and safety of adjuvant G250. The adjuvant treatment demonstrated a safe and well tolerated profile. However, G250 had no clinical benefit for the patients [169]. Antibodies were also proposed as imaging agents for CA-IX positive tumors, originally by Neri’s group [152]. Therefore, radiolabelled chimeric G250 was also tested as a valuable imaging agent for the diagnosis of patients with renal cell carcinoma. Zirconium-89- labeled girentuximab has been developed and entered in a phase II/III clinical trial (NCT02883153) to study the impact of the Zirconium-89-girentuximab in clinical management. Unfortunately, even if the study is completed, the results have not been published yet. Lastly, radioimmunotherapy with Lutetium 177–labeled girentuximab are also well tolerated in metastatic ccRCC patients [170].

## 5. Cocktail of Proton Exchanger Inhibitors as a Novel Therapeutic Approach against Cancer

Triggered from such encouraging results, we explored the hypothesis that PPIs could increase the effectiveness of CA-IX inhibitors against very malignant human melanoma cells, fully expressing the enzyme [52]. To this purpose, human melanoma cells have been treated with potent CA-IX inhibitors, the sulfamates S4, and *p*-nitrophenyl derivative FC9-399A (selective ureido-sulfamate derivatives), in combination with lansoprazole [52]. First of all, we observed that treatment with these CA-IX inhibitors induced a slight but significant inhibition of melanoma cell growth, which was strongly impaired under acidic conditions, typical of malignant cell growth [52]. We postulated that the impairment of CA-IX inhibitor activity was probably due to their neutralization by protonation outside the cells induced by acidity. When we compared the combination treatment at suboptimal doses (lansoprazole followed by one of the two CA-IX inhibitors), we observed a more efficient and significantly increased tumor cell growth inhibition, and a straightforward cytotoxic effect against metastatic melanoma cells, compared to each single agent [52]. We also demonstrated that the effect of combined treatments was not due to changes in the CA-IX protein expression, but rather probably to induction of an inhibition in CA-IX activity [52]. The pre-treatment of human melanoma cells with lansoprazole significantly improved the antitumor effect of CA-IX inhibitors, probably because lansoprazole, fully active in acidic conditions, induced an alkalinization of the tumor extracellular environment, in turn leading to stronger and the greatest activation and effectiveness of CA-IX inhibitors. Therefore, our results provided the first evidence that combinations of lansoprazole with two different CA-IX inhibitors were more effective than single treatments in inhibiting cell proliferation and inducing cell death in human melanoma cells. These results were supported by another recent study where we combined the proton pump inhibitor lansoprazole with the inhibitor of the reverse transcriptase, Efavirenz, in order to target in a unique treatment two new oncotypes, i.e., proton pumps and reverse transcriptase [171]. Again, the results clearly showed that pre-treatment of human melanoma cells with the proton pump inhibitor lansoprazole significantly improved the Efavirenz antitumor effect [171]. These two recent works of ours highlighted, for the first time, that the combination of proton pump and CA-IX/reverse transcriptase inhibitors possess a more efficient antitumor action compared to single treatments. Actually, they represent the first attempt aimed at combining either different proton exchangers inhibitors or a proton exchanger with the reverse transcriptase inhibitor in a unique antitumor approach, with conceivably more effectiveness and less toxicity.

Altogether, the results of these two studies clearly supported the hypothesis that treatment aimed at buffering the acidic tumor microenvironment, through the use of PPIs, that contrary to other drugs are activated in acidic conditions, may improve the effectiveness of CA-IX and reverse transcriptase inhibitors, significantly improving their antitumor effects. Thus, they open the way to novel and alternative antitumor strategies that are more specific, and probably less toxic for tumor patients. Of course, clinical trials obtained with lansoprazole followed by CA-IX or reverse transcriptase inhibitors are needed in order to further support the use of combination therapies that these studies were proposing.

## 6. Conclusions

Despite the great efforts of the scientific community in finding proper treatments for cancer, the responsiveness of human tumors to chemotherapy has not changed in the last decades, and resistance or refractoriness to chemotherapeutic drugs has become a key problem in the therapy of tumor patients and still remains unsolved. Therefore, novel antitumor strategies, which are more specific and probably less toxic, have become an urgent medical need. During the last decades, tumor metabolism and microenvironmental acidity are increasingly considered important determinants of tumor progression and drug resistance. Therefore, the mechanisms controlling the acidic pH of solid tumors have been proposed as selective and specific therapeutic targets in setting up novel antitumor strategies. Proton exchangers, whose expression and activity are upregulated by hypoxia and acidity, have gained attention in the last years, due to their crucial function in determining the acidification of the tumor microenvironment. In particular, V-ATPases and CA-IX represented interesting targets for the development of novel approaches in anticancer therapy. For these reasons, several V-ATPases and CA-IX inhibitors have been tested for their antitumor activity. Investigations reported that several inhibitors of the tumor-associated carbonic anhydrase isoform IX, and of V-ATPases, had a clear antineoplastic action, and may be useful to be combined and tested for developing novel antitumor therapies. We tested combinations of lansoprazole, targeting proton pumps, followed by CA-IX inhibitors, and observed that combined treatments, while inducing the alkalinization of tumor microenvironment, led to an increased effectiveness of CA-IX inhibitors against very malignant human melanoma cells [52]. The same effect was observed combining lansoprazole with the inhibitor of the reverse transcriptase, another important hallmark of cancers [171]. These results clearly supported the hypothesis that an approach aimed at targeting tumor extracellular acidity, combining two or more proton exchanger inhibitors with different antitumor actions, may open the way to the development of innovative and alternative antitumor strategies that are more specific, effective, and hopefully less toxic for tumor patients. Of course, data from clinical trials obtained through combination of PPIs with CA-IX inhibitors are needed, in order to further support the use of proton pump and CA-IX inhibitor combination therapies, and to translate these results to the patients. Finally, the results of these studies give support to new investigations aimed at the setting up of either hybrids or combined molecules containing both proton pump and CA-IX inhibitors.
